# Evaluating the Effect of Lymph Node Status on Survival in Large Colon Cancer

**DOI:** 10.3389/fonc.2018.00602

**Published:** 2018-12-11

**Authors:** Qi Liu, Dakui Luo, Qingguo Li, Ji Zhu, Xinxiang Li

**Affiliations:** ^1^Department of Colorectal Surgery, Fudan University Shanghai Cancer Center, Shanghai, China; ^2^Department of Oncology, Shanghai Medical College, Fudan University, Shanghai, China; ^3^Department of Radiation Oncology, Fudan University Shanghai Cancer Center, Shanghai, China

**Keywords:** tumor size, node-negative, colon cancer, survival, SEER

## Abstract

**Objective:** This study aimed to investigate the effect of lymph node status on survival in large colon cancer.

**Methods:** In the first cohort, patients diagnosed with non-metastatic colon cancer (*N* = 176,834) were identified from the Surveillance, Epidemiology, and End Results (SEER) database between January 1988 and December 2005. Further analyses were conducted in the other cohort (*N* = 855) from the Fudan University Shanghai Cancer Center (FUSCC) database.

**Results:** In the SEER cohort, CSS differences increased as the tumor enlarged until a threshold tumor size group (tumor measuring 7–8 cm, *P* < 0.001) was reached, in which node positivity showed the maximum negative effect on CSS; multivariate Cox analyses showed that tumors measuring 7–8 cm presented a significant lower risk of cancer-specific mortality compared with those measuring 2–4 cm [hazard ratio (HR) = 1.087; 95% confidence interval (CI) = 1.014–1.165, *P* = 0.018]. In the FUSCC cohort, N0 tumors measuring 21–40 mm presented a higher risk of recurrence compared with those measuring 41–80 mm.

**Conclusions:** Mortality risk of node positivity increased as tumor enlarged until a threshold tumor size (tumor size of 7–8 cm) was reached, mainly resulting from larger tumors without lymph node involvement being a surrogate for biologically indolent colon cancer of tumor recurrence. Our study could provide both researchers and clinicians a better understanding of colon cancer biology.

## Novelty and Impact Statements

The present study revealed that mortality risk of node positivity increased as tumor enlarged until a threshold tumor size (tumor size of 7–8 cm) was reached, mainly resulting from larger tumors without lymph node involvement being a surrogate for biologically indolent colon cancer of tumor recurrence. Our study, if validated in other large ones, could provide both researchers and clinicians a better understanding of colon cancer biology.

## Introduction

Colon cancer is one of the most commonly diagnosed cancers among men and women (1). In many kinds of solid tumors, large tumor sizes are thought to be associated with worse prognosis (2–6). Similarly, the tumor size is an independent prognostic factor that negatively impacts survival in patients with colon cancer (7, 8). It is consistent with the traditional view that, when tumor size increases, tumor cells have more potential to spread and be involved in lymph node metastasis (9). With regard to lymph node positivity, the tumor size is associated with significantly worse survival compared with lymph node negativity and plays a prognosis and clinical treatment guiding role in the American Joint Committee on Cancer TNM staging system (10, 11).

To the best of our knowledge, however, few studies have investigated lymph node status and tumor size in predicting survival in colon cancer. Only one study addressing the effect of tumor size involved in the lymph node status found that very small tumors would represent more aggressive malignancies with a distinct biology compared with larger tumors in lymph node-positive colon cancers, thereby challenging the traditional view that, large tumor size negatively impacts survival (12).

The aforementioned findings inspired the investigation of colon cancer biology. In the present study, first a large population-based analysis was conducted to explore the effect of lymph node positivity on cause-specific survival (CSS) among colon cancer patients with different tumor sizes from the Surveillance, Epidemiology, and End Results (SEER) database. Then, the relapse and distant metastasis pattern of tumor size were investigated in another cohort from the Fudan University Shanghai Cancer Center (FUSCC) database.

## Patients and Methods

### Patient Selection From the SEER Database

Shown as Supplementary Figure 1, the first cohort used in this study was from the SEER Program of the United States National Cancer Institute. A total of 176,834 patients diagnosed with non-metastatic colon cancer were identified between 1988 and 2005 for the initial analysis. These years were included because detailed tumor size was recorded starting from 1988 and a follow-up of 10 years was required to fulfill the study criteria (SEER follow-up ended in 2015).

The study endpoint used in the SEER cohort was CSS. The cause of death was categorized as colon cancer specific or non-colon cancer related, and CSS was calculated from the date of diagnosis to the date of colon cancer death. Patients who died of other causes were censored at the date of death.

### Patient Selection From the FUSCC Database

Patients (*N* = 855) diagnosed with non-metastatic colon cancer of the validation cohort was selected between January 2008 and December 2015 from the FUSCC database. All patients were identified by pathological examination after the operation, and patients with incomplete relevant data, such as TNM stage, tumor size, and tumor grade, were not included in this study. The clinicopathological characteristics of patients of the FUSCC cohort are shown in the Supplementary Table 2. This study was approved by the Ethical Committee and Institutional Review Board of FUSCC.

The outcomes of interest used in the FUSCC cohort were disease-free survival (DFS), relapse-free survival (RFS), and distant metastasis-free survival, which were calculated from the date of diagnosis to the date of the first event of recurrence, distant metastasis, or cause-specific death.

### Statistical Analyses

In the present study, the patient clinicopathological characteristics were compared between lymph node-positive and lymph node-negative groups using Pearson's chi-squared test. Several multivariate Cox proportional hazard models were constructed to test the differences in CSS. The survival curves in the present study were constructed using the Kaplan-Meier method, and a univariate survival difference was determined using the log-rank test. Statistical analysis was mainly performed using SPSS version 22 (SPSS Inc., IL, US); and two-sided *P* < 0.05 was considered statistically significant.

## Results

### Patient Characteristics of the SEER and FUSCC Cohorts

In the SEER cohort, the median follow-up time among censored patients was 118 months, and 41,443 (23.4%) patients died of colon cancer at the end of the follow-up time. Supplementary Table 1 summarized the baseline demographic characteristics of patients by the lymph node status. Lymph node negativity was correlated with low T stage, adenocarcinoma histology, low tumor grade, white race, old age, and small tumor size (*P* < 0.001). The median follow-up time of the FUSCC cohort was 43 months. The clinicopathological characteristics of the patients were shown in the Supplementary Table 2.

### Effect of Lymph Node Status on CSS in Different Tumor Size Groups

**Figure 1:**
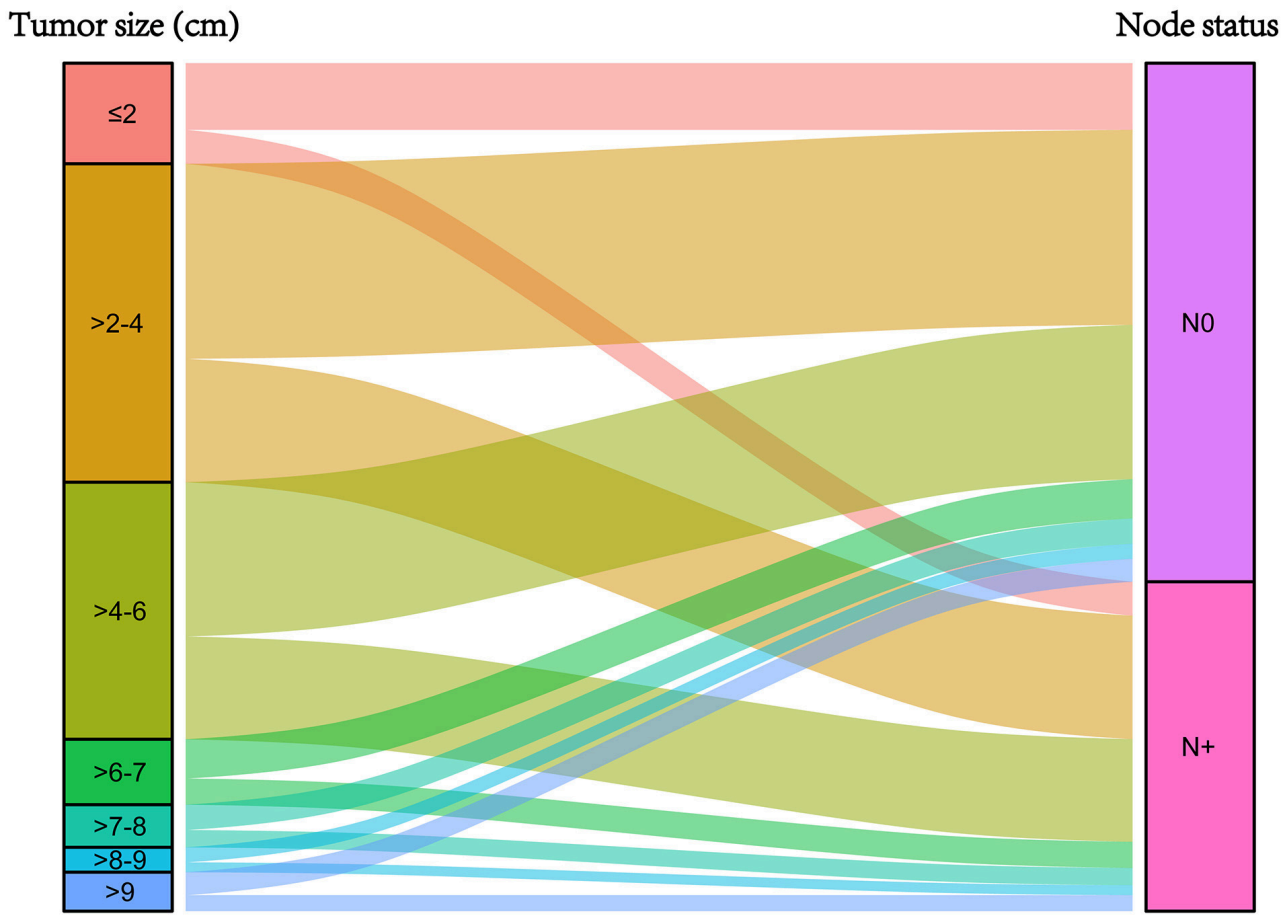
Graphical summary of tumor size and lymph node status, and their subgroup distribution.

**Figure 2:**
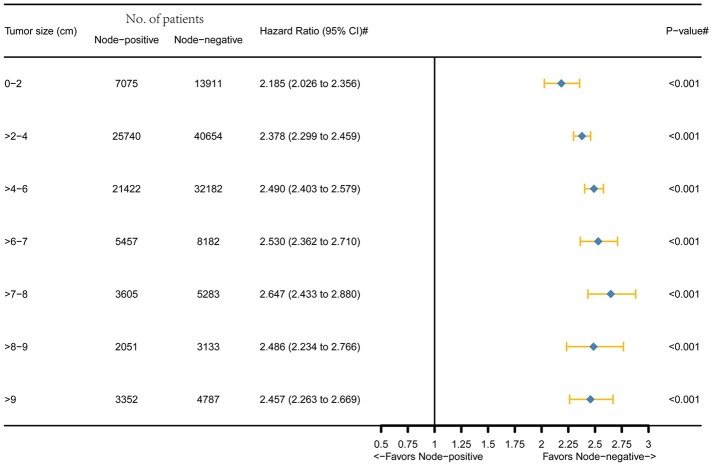
Hazard ratio comparing node-positivity and node-negativity according to the tumor size on CSS. (#) Multivariate analysis adjusted by T stage, histology, tumor grade, race, gender, tumor location, age at diagnosis, and year of diagnosis.

Figure 1 was the graphical summary of tumor size and lymph node status, and showed the distribution and associations of different tumor size and lymph node status. Several multivariate Cox proportional hazard models were conducted to test the effect of lymph node status on CSS in different tumor size groups after adjusting for T stage, histology, tumor grade, race, gender, tumor location, age at diagnosis, and year of diagnosis. The results are shown in Figure 2. All the survival differences between lymph node positivity and negativity in different tumor size groups were found to be significant (*P* < 0.001). Figure 2 reveals a pattern of increasing CSS differences as the tumor enlarged until a threshold tumor size group (tumor measuring 7–8 cm, *P* < 0.001) was reached, in which node positivity showed the maximum negative effect on CSS. After this, increasing tumor size was yet unexpectedly related to decreasing survival difference.

### Interaction Between Tumor Size and Lymph Node Status in CSS in the SEER Database

In the SEER cohort, multivariate Cox analyses were conducted after adjusting for T stage, histology, tumor grade, race, gender, tumor location, age at diagnosis, and year of diagnosis. A significant interaction was found between tumor size and lymph node status in determining CSS (*P* < 0.001; Table 1). In patients with lymph node-negative cancer, tumors measuring 7–8 cm presented a significant lower risk of cancer-specific mortality compared with those measuring 2–4 cm [hazard ratio (HR) = 1.087, 95% confidence interval (CI) = 1.014–1.165, using a tumor measuring 7–8 cm as reference]. Furthermore, a tumor measuring 7–8 cm showed similar CSS compared with that measuring < 2 cm (HR = 1.000, 95% CI = 0.918–1.089, using a tumor measuring 7–8 cm as reference) and experienced almost the lowest risk of cancer-specific mortality in groups of different tumor sizes. On the contrary, in patients with lymph node-positive cancer, a positive relationship was observed between smaller tumor size and increased CSS.

**Table 1:** Multivariate Cox regression analyses of CSS in SEER cohort.

**Variable**	**Overall**		**Pairwise**	
	**HR (95%CI)**	***P***	**HR (95%CI)**	***P***
**T stage**		< 0.001	…	…
T1	Reference			
T2	1.384 (1.298–1.475)	< 0.001		
T3	2.717 (2.567–2.876)	< 0.001		
T4a	3.892 (3.657–4.143)	< 0.001		
T4b	7.257 (6.812–7.731)	< 0.001		
**Histology**		< 0.001	…	…
Adenocarcinoma	Reference			
Mucinous adenocarcinoma	0.935 (0.903–0.967)	< 0.001		
Signet ring cell carcinoma	1.462 (1.351–1.581)	< 0.001		
**Tumor grade**		< 0.001	…	…
Grade I	Reference			
Grade II	1.081 (1.039–1.125)	< 0.001		
Grade III	1.292 (0.238–1.349)	< 0.001		
Grade IV	1.270 (1.137–1.419)	< 0.001		
Unknown	1.215 (1.143–1.291)	< 0.001		
**Race**		< 0.001	…	…
White	Reference			
Black	1.316 (1.276–1.357)	< 0.001		
Other	0.908 (0.874–0.944)	< 0.001		
Unknown	0.352 (0.215–0.574)	< 0.001		
**Gender**	0.918 (0.900–0.936)	< 0.001	…	…
Male	Reference			
Female	0.918 (0.900–0.936)			
**Tumor location**		< 0.001	…	…
Cecum	Reference			
Ascending colon	0.949 (0.912–0.982)	0.001		
Hepatic flexure	1.016 (0.973–1.061)	0.479		
Transverse colon	0.947 (0.912–0.982)	0.003		
Splenic flexure	1.054 (1.003–1.106)	0.036		
Descending colon	1.049 (1.005–1.095)	0.028		
Sigmoid Colon	1.110 (1.081–1.139)	< 0.001		
**Age at diagnosis (y)**		< 0.001	…	…
≤ 70	Reference			
>70	1.516 (1.485–1.546)			
**Year of diagnosis**		< 0.001	…	…
1988–1992	Reference			
1993–1996	0.950 (0.919–0.981)	< 0.001		
1997–2001	0.918 (0.892–0.945)	< 0.001		
2002–2005	0.864 (0.839–0.889)	< 0.001		
**Tumor size and node status**		< 0.001	…	…
≤ 2 cm, N0	Reference		1.000 (0.918–1.089)	0.996
>2–4 cm, N0	1.087 (1.023–1.154)	0.007	1.087 (1.014–1.165)	0.018
>4–6 cm, N0	1.068 (1.004–1.136)	0.038	1.068 (0.996–1.145)	0.064
>6–7 cm, N0	1.035 (0.959–1.117)	0.375	1.035 (0.954–1.124)	0.409
>7–8 cm, N0	1.000 (0.918–1.089)	0.996	Reference	
>8–9 cm, N0	1.080 (0.979–1.190)	0.123	1.080 (0.975–1.196)	0.139
>9 cm, N0	1.134 (1.042–1.233)	0.003	1.134 (1.037–1.239)	0.006
≤ 2 cm, N+	2.122 (1.972–2.283)	< 0.001	2.122 (1.952–2.307)	< 0.001
>2–4 cm, N+	2.589 (2.440–2.748)	< 0.001	2.590 (2.420–2.771)	< 0.001
>4–6 cm, N+	2.622 (2.468–2.786)	< 0.001	2.623 (2.450–2.807)	< 0.001
>6–7 cm, N+	2.628 (2.447–2.821)	< 0.001	2.628 (2.433–2.839)	< 0.001
>7–8 cm, N+	2.700 (2.500–2.916)	< 0.001	2.697 (2.456–2.962)	< 0.001
>8–9 cm, N+	2.696 (2.467–2.947)	< 0.001	2.701 (2.487–2.933)	< 0.001
>9 cm, N+	2.915 (2.699–3.149)	< 0.001	2.916 (2.685–3.166)	< 0.001

### Evaluating Previous Findings in the FUSCC Cohort

Considering that the SEER database did not provide any information such as tumor recurrence and chemotherapy, the findings from the SEER cohort should be interpreted cautiously. Then, the findings in 855 patients diagnosed with non-metastatic colon cancer between January 2008 and December 2015 from the FUSCC database were evaluated. In Table 2, the results of the multivariate Cox analysis showed that a tumor measuring 41–80 mm had better DFS compared with that measuring 21–40 mm among patients with lymph node-negative cancer (HR = 0.654, 95% CI = 0.375–1.142, *P* = 0.135, using an N0 tumor measuring 21–40 mm as reference). On the contrary, in patients with lymph node-positive cancer, tumors measuring 21–40 mm had better DFS compared with those measuring 41–80 mm (HR = 0.836, 95% CI = 0.555–1.258, *P* = 0.390, using an N+ tumor measuring 41–80 mm as reference). However, the differences in both patients with lymph node-negative and lymph node-positive cancer did not achieve statistical significance, which might account for relatively small sample size (*n* = 479) and short follow-up time (43 months). The DFS curves using the Kaplan-Meier method validated the aforementioned results (Supplementary Figure 2), both of which were consistent with the findings in the SEER cohort.

**Table 2:** Multivariate Cox regression analyses of CSS in FUSCC cohort.

**Variable**	**Overall**		**Pairwise**	
	**HR (95%)**	***P***	**HR (95%)**	***P***
**Tumor location**		0.323	…	…
Right colon	Reference			
Transverse colon	0.829 (0.396–1.739)	0.621		
Left colon	1.022 (0.631–1.655)	0.930		
Sigmoid colon	0.715 (0.488–1.047)	0.084		
**Age (years)**		0.726	…	…
≤ 70	Reference			
>70	1.082 (0.696–1.684)			
**Year of diagnosis**		0.643	…	…
2008–2011	Reference			
2012–2015	1.091 (0.755–1.576)			
**Gender**		0.900	…	…
Male	Reference			
Female	0.979 (0.705–1.361)			
**Neoadjuvant chemotherapy**		0.766	…	…
No	Reference			
Yes	0.872 (0.353–2.153)			
**Adjuvant chemotherapy**		0.044	…	…
No	Reference			
Yes	0.649 (0.426–0.989)			
**Tumor grade**		0.743	…	…
Well/moderately differentiated^*^	Reference			
Poorly differentiated/undifferentiated^*^	1.067 (0.726–1.567)			
**Histology**		0.525	…	…
Adenocarcinoma	Reference			
Mucinous adenocarcinoma/ signet ring cell carcinoma	1.153 (0.743–1.791)			
**T stage**		0.089	…	…
T1	Reference			
T2	1.337 (0.168–10.648)	0.784		
T3	2.347 (0.318–17.325)	0.403		
T4	3.019 (0.408–22.339)	0.279		
**Tumor size and nodal stage**		< 0.001	…	…
21–40 mm, N0	Reference		0.446 (0.263–0.756)	0.003
41–80 mm, N0	0.654 (0.375–1.142)	0.135	0.292 (0.180–0.472)	< 0.001
21–40 mm, N+	1.875 (1.120–3.140)	0.017	0.836 (0.555–1.258)	0.390
41–80 mm, N+	2.243 (1.322–3.807)	0.003	Reference	

Well/moderately differentiated*: *: including well differentiated, well-moderately differentiated and well-moderately differentiated*.

Poorly differentiated/undifferentiated*: *: including poorly-moderately differentiated poorly differentiated, and undifferentiated*.

**Figure 3:**
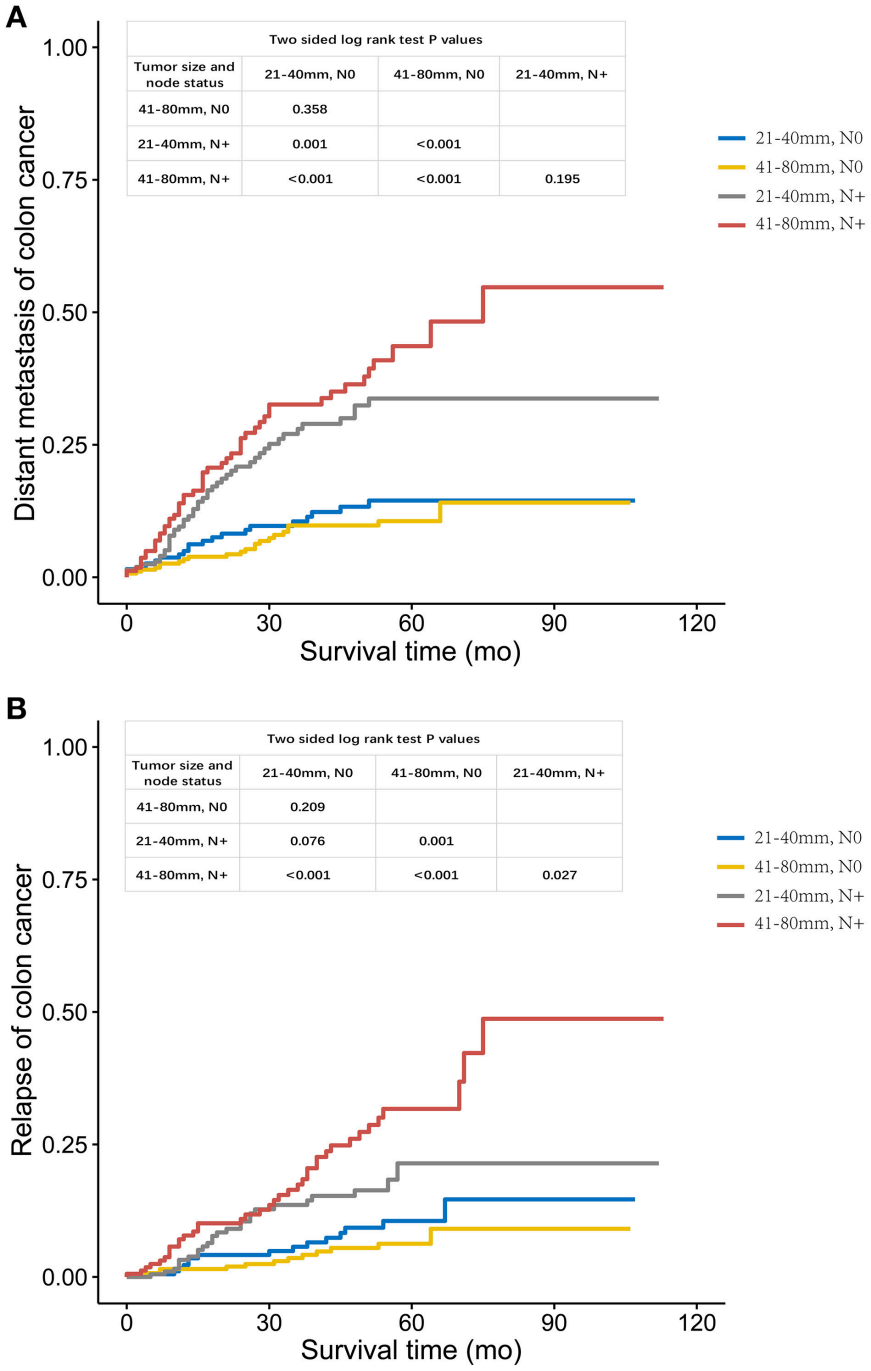
**(A)**. Distant metastasis and **(B)**. Relapse curves using the Kaplan-Meier method in FUSCC cohort.

Then, the relapse and distant metastasis pattern of large tumors were investigated. Among patients with lymph node-negative cancer, tumors measuring 21–40 mm had a similar risk of distant metastasis compared with those measuring 41–80 mm (log-rank *P* = 0.358, Figure 3A). However, in the case of RFS, tumors measuring 21–40 mm presented a high risk of recurrence compared with those measuring 41–80 mm, but the difference did not achieve statistical significance (log-rank *P* = 0.209, Figure 3B), which might account for relatively small sample size (*n* = 479) and short follow-up time (43 months).

In case of tumors measuring 21–40 mm, the increased risk of distant metastasis was obviously higher with node positivity compared with tumor recurrence (log-rank *P* = 0.001 for distant metastasis, Figure 3A; *P* = 0.076 for tumor recurrence, Figure 3B). Yet, increased risk of both distant metastasis and recurrence in larger tumors (41–80 mm) was obvious with lymph node positivity (log-rank *P* < 0.001 for distant metastasis, Figure 3A; *P* < 0.001 for tumor recurrence, Figure 3B).

## Discussion

In the present study, first we revealed a pattern of increasing CSS differences as the tumor enlarged until a threshold tumor size measuring 7–8 cm was reached, in which node positivity showed the maximum negative effect on CSS. After this, increasing tumor size was yet unexpectedly related to decreasing survival difference. This interesting phenomenon indicated that the interaction of lymph node status and tumor size was not monotonic and tumors measuring 7–8 cm presented the highest risk of cancer-specific mortality if involved in lymph node positivity. Multivariate Cox analyses then showed that, in the context of lymph node-negativity, larger tumors (7–8 cm) presented a significantly lower risk of cancer-specific mortality compared with smaller ones (2–4 cm). Furthermore, tumors measuring 7–8 cm showed a similar CSS compared with those measuring < 2 cm. However, in the lymph node-positive group, monotonically increased CSS was observed as the tumor enlarged. Consequently, the piecewise and regular relationship of lymph node positivity on survival with increasing tumor size was attributed to the non-monotonical effect of tumor size on CSS among lymph node-negative tumors.

In addition, the findings of the present study in the FUSCC cohort furtherly showed that N0 tumors with larger tumor size (41–80 mm) presented a lower risk of tumor recurrence, which could account for the better CSS in this subgroup. That meant node positivity showing the maximum negative effect on CSS in tumor size measuring 7–8 cm was probably because of the lower risk of tumor recurrence in larger colon cancer without node involvement.

Traditionally, cancer gains the ability to metastasize as it grows to a larger size (13). Yet, recent studies have suggested that metastasis can occur at an early point of tumor progression (14–17). In 2016, Muralidhar et al. (12) found that too small tumor size might be a surrogate for biologically aggressive disease and predict for increased colon cancer-specific mortality compared with larger tumors in the setting of lymph node involvement. However, the present study found that larger N0 colon cancer might be a surrogate for the biologically indolent disease, where metastases pathways had less advantage than those resulting in “reattachment in or at the primary site” according to the “self-seeding” concept (13). The findings coupled with those of Muralidhar et al. (12) supported and added a growing body of evidence to the view that metastasis that occurred at an early point of tumor progression rather than the accumulated metastatic ability during tumor evolution likely determined poor prognosis.

The present study showed the piecewise relationship between tumor size and CSS among node-negative colon cancer, resulting in the piecewise relationship between lymph node status and CSS in different tumor size groups. Tumors measuring >7–8 cm had better CSS compared with those measuring >2–4, >4–6, >6–7, >8–9, and >9 cm, and had a similar CSS compared with those measuring ≤ 2 cm. According to the linear progression model of cancer progression (17), cancer cells could pass through multiple successive rounds of mutation, and selection for competitive fitness in the context of primary and larger tumors was linked to the more clonal expansion of fully malignant clones. It could be found in the results of the SEER cohort that 66.3% of tumors measuring < 2 cm were lymph node negative, whereas 61.2% of those measuring >2–4 were lymph node negative, indicating that 7.6% of the present node-negative tumors measuring < 2 cm would become node-positive disease if they continued to grow, which was consistent with the linear progression model of cancer progression that larger tumors were more likely to present clonal expansions and mutations. However, a reasonable explanation for the larger tumors without lymph node positivity was that they could experience less clonal expansion and mutation, contributing to the failure of lymph node involvement, and why this subset of larger tumors experience less clonal expansion and mutation need future investigation. A further analysis in the FUSCC cohort indicated that the better survival of larger tumors than smaller ones was mainly accounted for by the lower risk of tumor recurrence, as N0 tumors measuring 41–80 mm presented a significantly lower risk of tumor recurrence compared with those measuring 21–40 mm, suggesting that larger tumors without node involvement might experience less clonal expansions and mutations that contributing to tumor recurrence and node involvement together. These clonal expansions and mutations need to be identified in further studies.

The results were remarkable in terms of both basic research and clinical significance. The better DFS and CSS in node-negative larger tumors pointed out a direction for the follow-up studies focusing on gene mutations and clonal expansions contributing to tumor recurrence in node-negative tumors. The highest risk of colon cancer-specific mortality with lymph node positivity in tumors measuring 7–8 cm, coupled with the fact that tumor size was basically < 8 cm in clinical practice of colon cancer (92.5% in SEER cohort), indicated that node-negativity showed increased survival improvement compared with node involvement as tumor enlarged in almost all the colon cancer.

This study had some limitations. First, it did not include some prognostic factors such as microsatellite instability, BRAF V600E mutation, and carcinoembryonic antigen level, introducing biases to some extent (18–20). Second, due to the relatively small sample size (*n* = 855) and short follow-up time (43 months) of the FUSCC cohort, some DFS differences in multivariate Cox analyses and RFS differences between N0 tumors measuring 21–40 and 41–80 mm did not achieve statistical significance. Then, the different outcomes of interest in SEER (CSS) and FUSCC (RFS) cohorts resulted into the fact that the comparability of the two patient cohorts was narrowed and could introduce unaccounted biases. Finally, two cohorts in this study were both retrospective rather than prospective. These findings still need to be validated in other prospective cohorts.

In conclusion, mortality risk of node positivity increased as tumor enlarged until a threshold tumor size (tumor size of 7–8 cm) was reached, mainly resulting from larger tumors without lymph node involvement being a surrogate for biologically indolent colon cancer of tumor recurrence. Larger tumors without node involvement might experience less clonal expansions and mutations that contributing to tumor recurrence and node involvement together, and these findings could elicit further studies to identify these clonal expansions and mutations. It was also of great clinical significance as it showed that larger node-positive colon cancer deserved more attention, mainly to reduce the risk of postoperative recurrence.

## Ethics Statement

The study was approved by the Ethical Committee and Institutional Review Board of the Fudan University Shanghai Cancer Center.

## Author Contributions

XL and JZ conceived this study. QLiu and DL improved the study design and contributed to the interpretation of results. QLiu and JZ collected the data. QLiu performed data processing and statistical analysis. QLiu and QLi wrote the manuscript. XL, JZ, and QLi revised the manuscript and approved the final version.

### Conflict of Interest Statement

The authors declare that the research was conducted in the absence of any commercial or financial relationships that could be construed as a potential conflict of interest.

## Supplementary Material

Supplementary Figure 1 Schematic representation of patient population selected from SEER database.

Supplementary Figure 2 DFS curves using the Kaplan-Meier method in FUSCC cohort.

Supplementary Table 1 Comparison of baseline characteristics by the lymph node status in SEER cohort.

Supplementary Table 2 Comparison of baseline characteristics by lymph node status in FUSCC cohort.
