# Supplementary figures and images for: Evaluating the Effect of Lymph Node Status on Survival in Large Colon Cancer
Source: Front Oncol. 2018 Dec 11;8:602. doi: 10.3389/fonc.2018.00602 (PMC6298250; doi:10.3389/fonc.2018.00602)

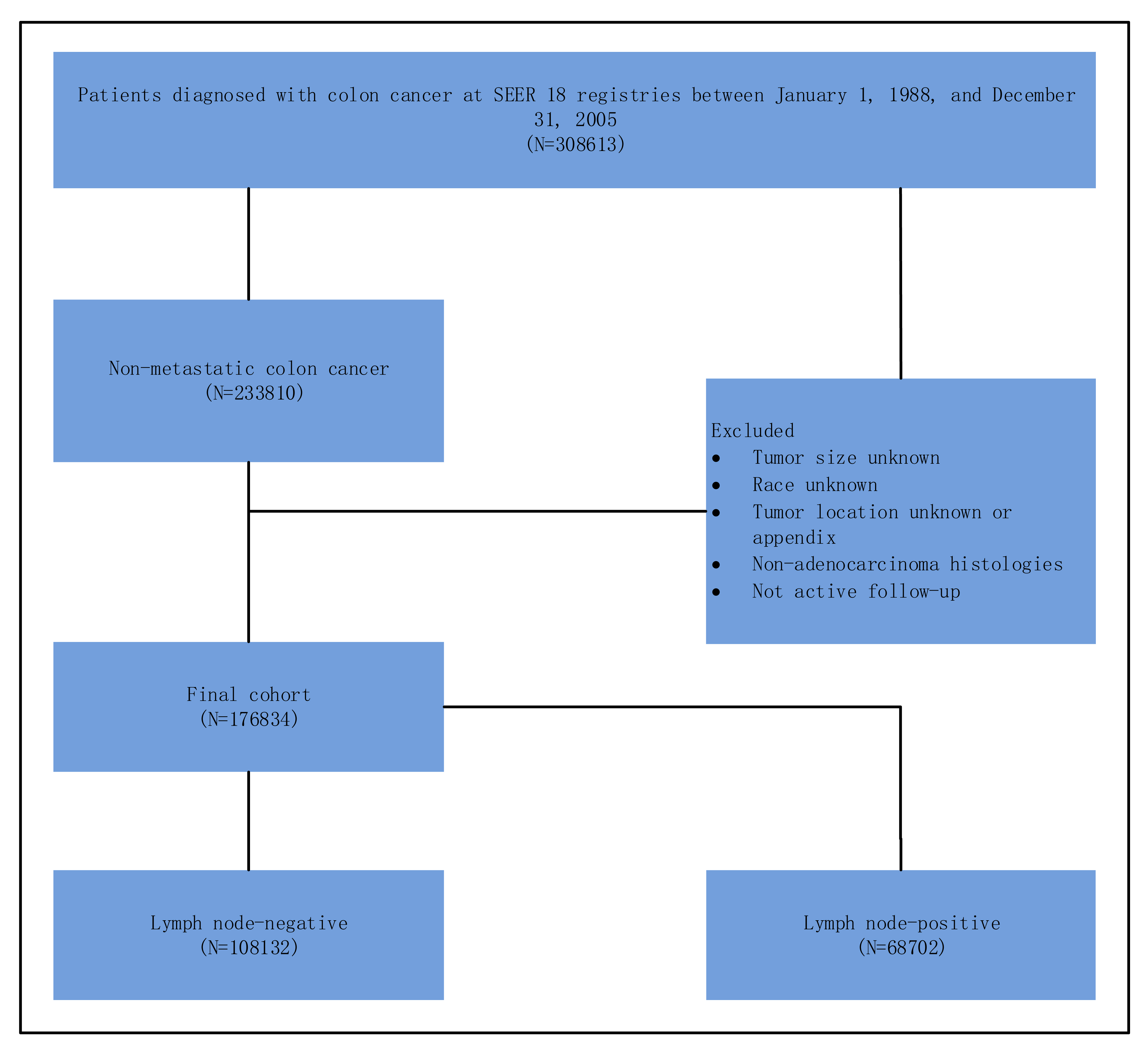

Supplement: Supplementary Figure 1 — Schematic representation of patient population selected from SEER database. [file Image_1.JPEG]

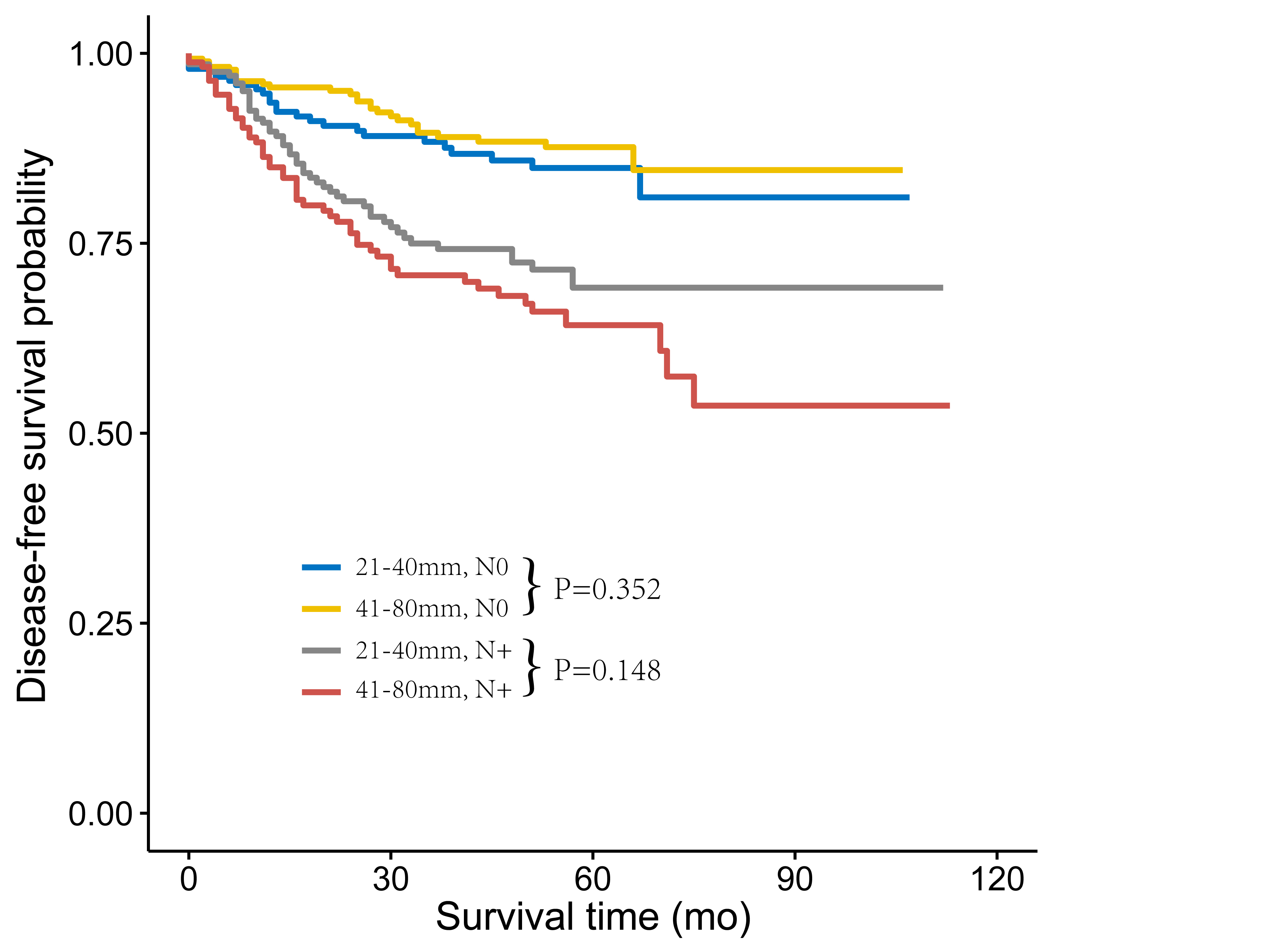

Supplement: Supplementary Figure 2 — DFS curves using the Kaplan-Meier method in FUSCC cohort. [file Image_2.JPEG]
