# Supplementary material for: Evaluating the Effect of Lymph Node Status on Survival in Large Colon Cancer
Source: Front Oncol. 2018 Dec 11;8:602. doi: 10.3389/fonc.2018.00602 (PMC6298250; doi:10.3389/fonc.2018.00602)
Supplement: Supplementary Table 1 — Comparison of baseline characteristics by the lymph node status in SEER cohort. [file Data_Sheet_1.docx]

**Supplementary Table 1. Comparison of baseline characteristics by the lymph node status in SEER cohort**

| **Variable** | **No. of Patients (%)** | | ***P*** |
| --- | --- | --- | --- |
|  | **Node-negative**  **(N=108132)** | **Node-positive**  **(N=68702)** |  |
| **T stage** |  |  | <0.001 |
| **T1** | 11936 (11.0) | 5514 (8.0) |  |
| **T2** | 21878 (20.2) | 5488 (8.0) |  |
| **T3** | 62850 (58.1) | 44650 (65.0) |  |
| **T4a** | 7159 (6.6) | 7329 (10.7) |  |
| **T4b** | 4309 (4.0) | 5721 (8.3) |  |
| **Histology** |  |  | <0.001 |
| **Adenocarcinoma** | 98252 (90.9) | 61067 (88.9) |  |
| **Mucinous adenocarcinoma** | 9362 (8.7) | 6501 (9.5) |  |
| **Signet ring cell carcinoma** | 518 (0.5) | 1134 (1.7) |  |
| **Tumor grade** |  |  | <0.001 |
| **Grade I** | 11523 (10.7) | 4607 (6.7) |  |
| **Grade II** | 76456 (70.7) | 42472 (61.8) |  |
| **Grade III** | 15543 (14.4) | 17359 (25.3) |  |
| **GradeⅣ** | 553 (0.5) | 689 (1.0) |  |
| **Unknown** | 4057 (3.8) | 3575 (5.2) |  |
| **Race** |  |  | <0.001 |
| **White** | 91685 (84.8) | 56136 (81.7) |  |
| **Black** | 9504 (8.8) | 7161 (10.4) |  |
| **Other** | 9831 (6.3) | 5331 (7.8) |  |
| **Unknown** | 112 (0.1) | 74 (0.1) |  |
| **Gender** |  |  | 0.066 |
| **Male** | 51304 (47.4) | 32904 (47.9) |  |
| **Female** | 56828 (52.6) | 35798 (52.1) |  |
| **Tumor location** |  |  | <0.001 |
| **Cecum** | 27461 (25.4) | 17689 (25.7) |  |
| **Ascending colon** | 21301 (19.7) | 11858 (17.3) |  |
| **Hepatic flexure** | 7111 (6.6) | 3897 (5.7) |  |
| **Transverse colon** | 11555 (10.7) | 6852 (10.0) |  |
| **Splenic flexure** | 4566 (4.2) | 2987 (4.3) |  |
| **Descending colon** | 6788 (6.3) | 4380 (6.4) |  |
| **Sigmoid Colon** | 29350 (27.1) | 21039 (30.6) |  |
| **Age at diagnosis (y)** |  |  | <0.001 |
| **≤70** | 43038 (39.8) | 31879 (46.4) |  |
| **>70** | 65094 (60.2) | 36823 (53.6) |  |
| **Year of diagnosis** |  |  | <0.001 |
| **1988-1992** | 17000 (15.7) | 10917 (15.9) |  |
| **1993-1996** | 17424 (16.1) | 11624 (16.9) |  |
| **1997-2001** | 34087 (31.5) | 21399 (31.1) |  |
| **2002-2005** | 39621 (36.6) | 24762 (36.0) |  |
| **Tumor size (cm)** |  |  | <0.001 |
| **≤2** | 13911 (12.9) | 7075 (10.3) |  |
| **>2-4** | 40654 (37.6) | 25740 (37.5) |  |
| **>4-6** | 32182 (29.8) | 21422 (31.2) |  |
| **>6-7** | 8182 (7.6) | 5457 (7.9) |  |
| **>7-8** | 5283 (4.9) | 3605 (5.2) |  |
| **>8-9** | 3133 (2.9) | 2051 (3.0) |  |
| **>9** | 4787 (4.4) | 3352 (4.9) |  |

**Supplementary Table 2. Comparison of baseline characteristics by lymph node status in FUSCC cohort**

| **Characteristic** | **No. of Patients (%)** | | ***P*** |
| --- | --- | --- | --- |
|  | **Node-negative (N=479)** | **Node-positive (N=376)** |  |
| **Tumor location** |  |  | 0.232 |
| **Right colon** | 211 (44.1) | 144 (38.3) |  |
| **Transverse colon** | 33 (6.9) | 21 (5.6) |  |
| **Left colon** | 55 (11.5) | 48 (12.8) |  |
| **Sigmoid colon** | 180 (37.6) | 163 (43.4) |  |
| **Age (years)** |  |  | 0.507 |
| **≤70** | 397 (82.9) | 318 (84.6) |  |
| **>70** | 82 (17.1) | 58 (15.4) |  |
| **Year of diagnosis** |  |  | 0.168 |
| **2008-2011** | 136 (28.4) | 91 (24.2) |  |
| **2012-2015** | 343 (71.6) | 285 (75.8) |  |
| **Gender** |  |  | 0.897 |
| **Male** | 290 (60.5) | 226 (60.1) |  |
| **Female** | 189 (39.5) | 150 (39.9) |  |
| **Neoadjuvant chemotherapy** |  |  | 0.595 |
| **No** | 462 (96.5) | 360 (95.7) |  |
| **Yes** | 17 (3.5) | 16 (4.3) |  |
| **Adjuvant chemotherapy** |  |  | <0.001 |
| **No** | 209 (43.6) | 56 (14.9) |  |
| **Yes** | 270 (56.4) | 320 (95.1) |  |
| **Tumor grade** |  |  | 0.009 |
| **Well/moderately differentiated** | 369 (77.0) | 260 (69.1) |  |
| **Poorly differentiated/undifferentiated** | 110 (23.0) | 116 (30.9) |  |
| **Histology** |  |  | 0.868 |
| **Adenocarcinoma** | 397 (82.9) | 310 (82.4) |  |
| **Mucinous adenocarcinoma/ signet ring cell carcinoma** | 82 (17.1) | 66 (17.6) |  |
| **T stage** |  |  | <0.001 |
| **T1** | 18 (3.8) | 2 (0.5) |  |
| **T2** | 79 (16.5) | 23 (6.1) |  |
| **T3** | 209 (43.6) | 116 (30.9) |  |
| **T4** | 173 (36.1) | 235 (62.5) |  |
| **Tumor size (cm)** |  |  | <0.001 |
| **2-4** | 196 (40.9) | 207 (55.1) |  |
| **>4-8** | 283 (59.1) | 169 (44.9) |  |

Abbreviation: FUSCC, Fudan University Shanghai Cancer Center

Well/moderately differentiated**^*^**: including well differentiated, well-moderately differentiated and well-moderately differentiated

Poorly differentiated/undifferentiated**^*^**: including poorly-moderately differentiated poorly differentiated, and undifferentiated
